# Aging affects epidermal Langerhans cell development and function and alters their miRNA gene expression profile

**DOI:** 10.18632/aging.100501

**Published:** 2012-11-24

**Authors:** Ying-Ping Xu, Rui-Qun Qi, Wen-Bin Chen, Yu-Ling Shi, Zhi-Zhong Cui, Xing-Hua Gao, Hong-Duo Chen, Li Zhou, Qing-Sheng Mi

**Affiliations:** ^1^ Henry Ford Immunology Program, Henry Ford Health System, Detroit, MI 48202, USA; ^2^ Department of Dermatology, Henry Ford Health System, Detroit, MI 48202, USA; ^3^ Department of Preventive Veterinary Medicine, College of Animal Science and Veterinary Medicine, Shandong Agriculture University, Taian, China; ^4^ Department of Dermatology, NO. 1 Hospital, China Medical University, Shenyang, China; ^5^ Department of Internal Medicine, Henry Ford Health System, Detroit, MI 48202, USA

**Keywords:** Langerhans cells, aging, miRNAs, skin, dendritic cells

## Abstract

Immunosenescence is a result of progressive decline in immune system function with advancing age. Epidermal Langerhans cells (LCs), belonging to the dendritic cell (DC) family, act as sentinels to play key roles in the skin immune responses. However, it has not been fully elucidated how aging affects development and function of LCs. Here, we systemically analyzed LC development and function during the aging process in C57BL/6J mice, and performed global microRNA (miRNA) gene expression profiles in aged and young LCs. We found that the frequency and maturation of epidermal LCs were significantly reduced in aged mice starting at 12 months of age, while the Langerin expression and ability to phagocytose Dextran in aged LCs were increased compared to LCs from < 6 month old mice. The migration of LCs to draining lymph nodes was comparable between aged and young mice. Functionally, aged LCs were impaired in their capacity to induce OVA-specific CD4^+^ and CD8^+^ T cell proliferation. Furthermore, the expression of miRNAs in aged epidermal LCs showed a distinct profile compared to young LCs. Most interestingly, aging-regulated miRNAs potentially target TGF-β-dependent and non- TGF-β-dependent signal pathways related to LCs. Overall, our data suggests that aging affects LCs development and function, and that age-regulated miRNAs may contribute to the LC developmental and functional changes in aging.

## INTRODUCTION

Immunosenescence is a result of progressive and gradual decline in immune system function with advancing age. Associated with a generalized impairment of immune function, it is known that aged populations are increasingly susceptible to cutaneous viral and fungal infections as well as to skin cancers that are accompanied with immunological deficiencies [1]. The effect on the immune system due to aging involves both humoral and cell mediated immunity. Defects in cellular immunity include a decrease in absolute numbers of naïve T cells and alterations of T cell function [2]. Recent studies have shown that aging related defects in the immune system are not restricted to adaptive immunity but can also extend to the innate immune response. However, the impact of aging on innate immunity is far less clear than its impact on adaptive immunity.

There are many reports suggesting that different cellular components of the innate immune system such as neutrophils, natural killer (NK) cells, NKT, and macrophages, which are all important first lines of defense against bacterial and parasitic infections, are defective in aged mice and human [3-5]. DCs are professional antigen-presenting cells (APCs) that are important in the initial priming of naïve T cells and act as a bridge to link innate immunity and adaptive immunity. Several studies indicate that the number of DCs does not change with age [6, 7]. However, Schodel et al. found a decrease in circulating plasmocytoid DCs numbers but observed no change in the number of myloid DCs [8]. There is also some controversy regarding the capacity of aged DCs to stimulated T cells [8, 9]. Epidermal LCs are skin-resident DCs with a life cycle distinct from other types of DCs. They play a key role in the development of cutaneous immune responses. Previous studies have speculated that age-related changes in epidermal LCs frequency and/or migration patterns results in impaired immunosurveillance in human [10]. However, the precise effects of aging on LCs are still not well understood.

Aging is a multifactorial process where deterioration of organism functions is driven by distinct genetically encoded genes. To better understand the genetic component of aging, many studies have addressed the distinct gene and protein expression profiles in different aging cells. microRNAs (miRNAs), small non-coding RNAs, have recently emerged as key regulators of gene expression through mRNA degradation, and/or translation inhibition [11]. The ribonuclease III enzyme Dicer is required for the processing of mature miRNAs. Therefore, deletion of Dicer provides a good genetic tool to test the relevance of miRNAs in mammalian development. Using a mouse model with the tissue-specific deletion of Dicer, studies from our group and others indicate that lack of miRNAs by Dicer-deletion dramatically interrupts T and B cell development and function [12-14]. Interestingly, deletion of Dicer specifically in DCs only affects the homeostasis and function of LCs, but not other types of DCs. This suggests that miRNAs are required for normal LCs development and function [15]. Importantly, miRNAs are involved in aging-mediated T cell and macrophage defects [16, 17], and play an important role in aging process and aging-related diseases [18-20]. However, it is unclear whether aging affects miRNA gene expression in LCs, which may contribute to aging-mediated LC development and function.

In the current study, we systemically investigated the development and function of LCs during aging using C57BL/6J mice and performed miRNA expression profiles of LCs between aged and young mice. Our results demonstrate that the frequency and maturation of epidermal LCs were reduced in a stepwise fashion in aged mice, starting at 12 months old. Aged LCs exhibited a reduced ability to stimulate T cell proliferation. However, aged LCs migration toward skin draining lymph nodes was not altered compared to young LCs. Most important of all, genome-wide assessment of miRNA expression in epidermal LCs revealed a distinct aging-specific miRNA gene expression profile. Taken together, our findings further confirm the defects of LCs development and function, and suggest the involvement of miRNAs in aged LCs.

## RESULTS

### Decreased frequencies of epidermal LCs in aged mice

**Figure 1:**
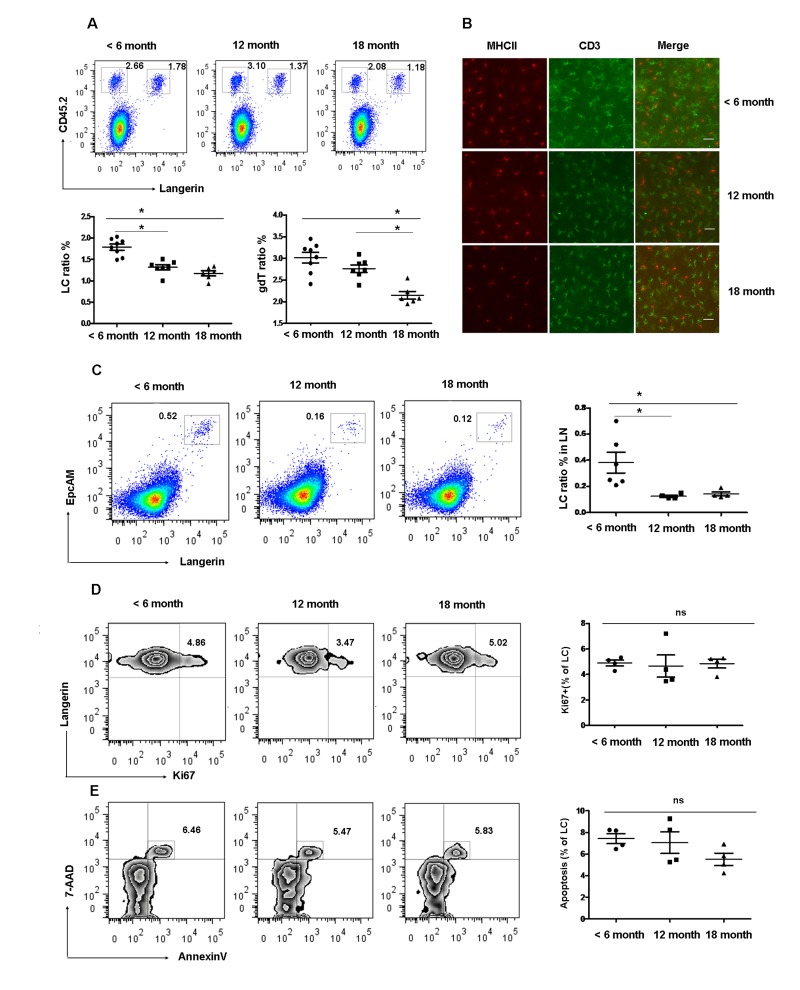
Gradual loss of epidermal LCs during aging development (**A**) and (**B**) Reduced epidermal LCs and DETCs during aging development. The number of Langerin^+^CD45.2^+^epidermal LCs and Langerin^−^CD45.2^+^ DETCs were analyzed by FACS (**A**). Data are shown as mean±SEM of 6-8 mice at each time point. MHCII^+^ LCs and CD3^+^ DETCs in the epidermis was assessed by imunofluorescence at each time point (B). Scale bar, 10μm, original magnification (200×). (**C**) Migrated LCs in draining LNs. Cells from LNs were stained by Anti-IAE, CD8, EpCAM, and Langerin. The EpCAM^+^Langerin^+^ migrated LCs were analyzed on gated CD8^−^IAE^+^ cells. Data are also shown as mean±SEMof 4-6 mice at each time point. *p < 0.05. (**D**) The kinetics of appearance of Ki67-labeled LCs in aged and young mice was determined by FACS analysis. (**E**) Apoptotic epidermal LCs were elucidated using Annexin-V antibody and the viable stain 7-AAD. Data are shown as mean ± SEM of 4 mice at each time point and representative of 2 independent experiments.

The density of LCs in the epidermis is known to decrease with age in mice [21]. However, the kinetics of LCs reduction with age has not been analyzed yet. To determine the effect of age on epidermal LCs homeostasis, we examined the frequency of skin LCs at different time points. The frequency of epidermal LCs (Langerin^+^CD45.2^+^) was 1.60 ± 0.09% in < 6 months old mice and was significantly reduced to 1.32 ± 0.02% in 12 months old mice and 1.13±0.10% in 18 months old mice. There was no significant difference between 12 months and 18 months old mice (Figure 1 A and B). In consistence with reduced epidermal LCs, the proportion of Langerin^+^EpCAM^+^ cells in the gated MHCII^+^CD8^−^ cells of LN, which represents the migrant epidermal LCs, decreased 2-3-fold in 12 and 18 months old mice compared with 6 months old mice (Figure 1 C). Interestingly, the frequency of dendritic epidermal T cells (DETC) was comparable between 6 and 12 month old mice but significantly decreased at 18 months (Figure 1 A and B). A previous study pointed out that the frequency of conventional DCs was reduced in 20-22 months of age mice [22]. Thus, the frequency of epidermal LCs is reduced earlier compared to other immune cells during the aging process. In subsequent studies, we used mice more than 12 months of age as aged mice for our LCs studies.

The markedly reduced frequency of LCs in aged mice could be explained by either a decrease in the LC proliferation, increased LC apoptosis, or by a decrease in the number of LCs progenitors. To study the first possibility, we preformed anti-Ki67 antibody staining as a way of measuring the kinetics of LCs turnover. We found no difference in the lifespan of LCs in aged and young mice (Figure 1 D). Furthmore LCs from aged mice exhibited normal rates of apoptosis as assessed by Annexin V and 7-AAD staining (Figure 1 E) compared to young LCs. This result further confirms the previous observation by Sprecher et al., suggesting that the decreased LCs density in aged mice could result from a deficiency in local LCs progenitors [21].

### Epidermal LCs from aged mice exhibit less ability to up-regulate MHCII and co-stimulatory molecules during in vitro maturation

**Figure 2:**
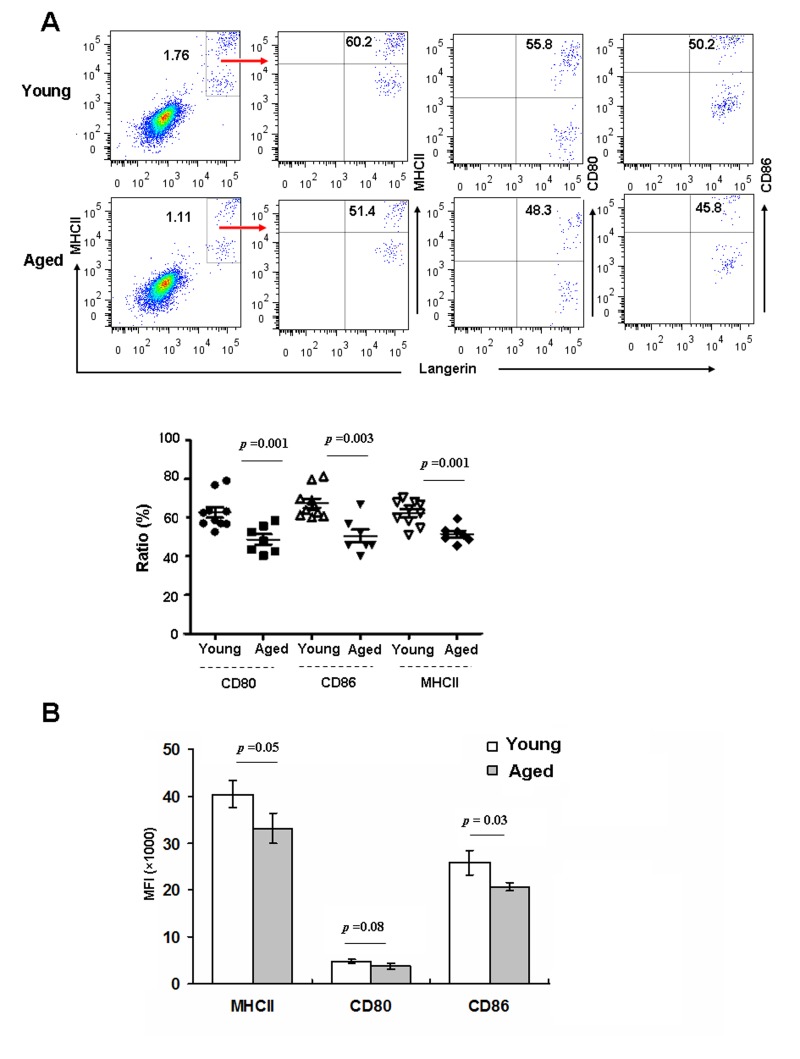
Aging inhibited LC maturation Epidermal cells were cultured in RPMI for 48h, and then stained with anti-Langerin, MHCII, CD80, CD86 antibodies, and analyzed by flow cytometry. The expression of MHCII and co-stimulatory molecules was determined by the ratio (**A**) and mean fluorescence intensity (MFI) (**B**). Data are shown as mean ± SEM of 6-10 mice each group.

The initiation, execution, and sustainment of T cell immunity by DCs require cell-cell contact mediated by costimulatory molecules on the DCs [23]. Immature LCs in the epidermis mature in response to various stimuli, and then migrate to draining LNs to prime T cells [24]. Hence, we investigated whether aging can affect epidermal LCs maturation *in vitro*. Epidermal cells derived from aged and young mice were cultured for 48h *in vitro*, and then stained with anti-CD80, CD86 and MHC-II, and assessed by FACS. As shown in Fig 2, the frequencies of these maturation markers were reduced on aged LCs (Figure 2 A), and expression levels of CD86 and MHCII based on MFI were also reduced (Figure 2 B). Thus, aged LCs have a lower ability to up-regulate MHCII and co-stimulatory molecules upon *in vitro* maturation.

### Aging LCs have an increased ability to phagocytize antigen but do not display altered allergen-induced migration

**Figure 3:**
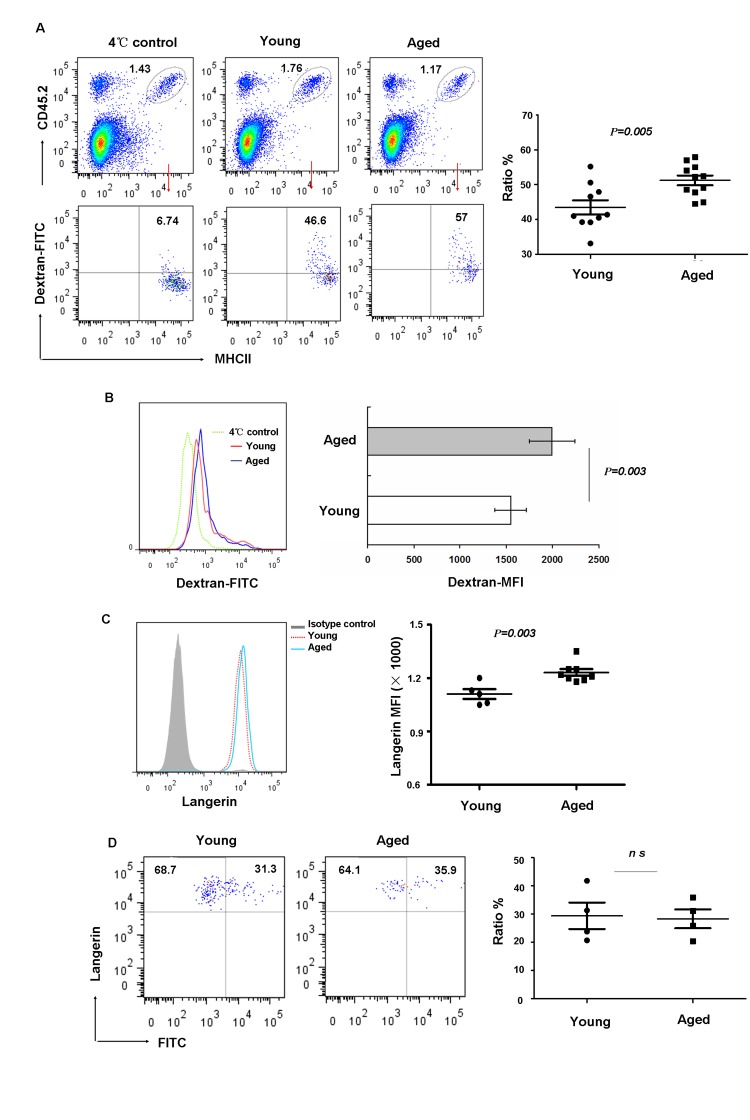
Aging impaired LC phagocytic ability but did not affect migration *in vivo* (**A-C**) Epidermal cells were incubated with 0.25 mg/ml FITC-Dextran for 45min at 4°C (control) or 37°C, and then stained with anti-MHCII and anti-CD45.2 antibodies. The percentage of FITC^+^ cells in LCs (CD45.2^+^MHCII^+^ cells) was determined (**A**). The geometric mean fluorescence of FITC expression in LCs was also shown (B) and the expression of Langerin in aging LCs was shown (**C**).(**D**) Aged LC migration. Mice were painted with 200μl 5mg/ml FITC in acetone/dibutylphthalate on the abdomen. LN cells collected 24hrs later were stained with anti-CD8, anti-Langerin and anti-EpCAM antibodies. The ratio of migrated FITC^+^ LCs was analyzed on gated Langerin^+^EpCAM^+^ LCs. Data are representative of 2 independent experiments, 4 mice were analyzed in each experiment. ns represents non-statistical significance.

LCs act as sentinels for contact with various environmental antigens. They capture and process exogenous antigens and migrate to the skin LNs where they activate T lymphocytes. Therefore we investigated whether aging can affect LC phagocytosis and migration. Freshly isolated LCs from aged and young mice were incubated *in vitro*, for 45 minutes at 37°C or 4°C (control) with Dextran-FITC and then stained with anti-MHCII and anti-CD45.2 mAb. As shown in Figure 3, LCs from aged mice had increased phagocytic capacity compared to that from young mice based on the ratio of FITC-positive (Figure 3 A) or mean fluorescence intensity expression levels (Figure 3 B). This is the additional example of hyper-functions associated with aging as discussed recently [25, 26]. Previous studies [27, 28] suggested that Langerin is the main molecular component of Birbeck granules and plays a substantial role in antigen uptake. Thus, we further investigated if aging affects Langerin expression. Interestingly, consistent with increased antigen uptake, we found increased Langerin expression in aged LCs (Figure 3 C).

To test whether or not the migratory capability of epidermal LCs was affected in vivo by aging, FITC was applied to the belly of young and aged mice as a tracer. After 24h, LCs from inguinal LN were harvested and the frequency of FITC^+^LCs was analyzed. Recent studies revealed the presence of at least 3 distinct skin DC subsets in LN based on the differential expression of CD8, EpCAM and Langerin [29], with migrant epidermal LCs being Langerin^+^EpCAM^+^ in the gated MHCII^+^CD8^−^ population. As shown in Figure 3 D, the frequencies of Langerin^+^FITC^+^LCs were comparable between aged and young mice. Together, these data indicated that phagocytic capacity increased with aging, however, the migration ability of aged LCs did not alter compared to young mice.

### Aging impaired LCs-mediated T cell proliferation

**Figure 4:**
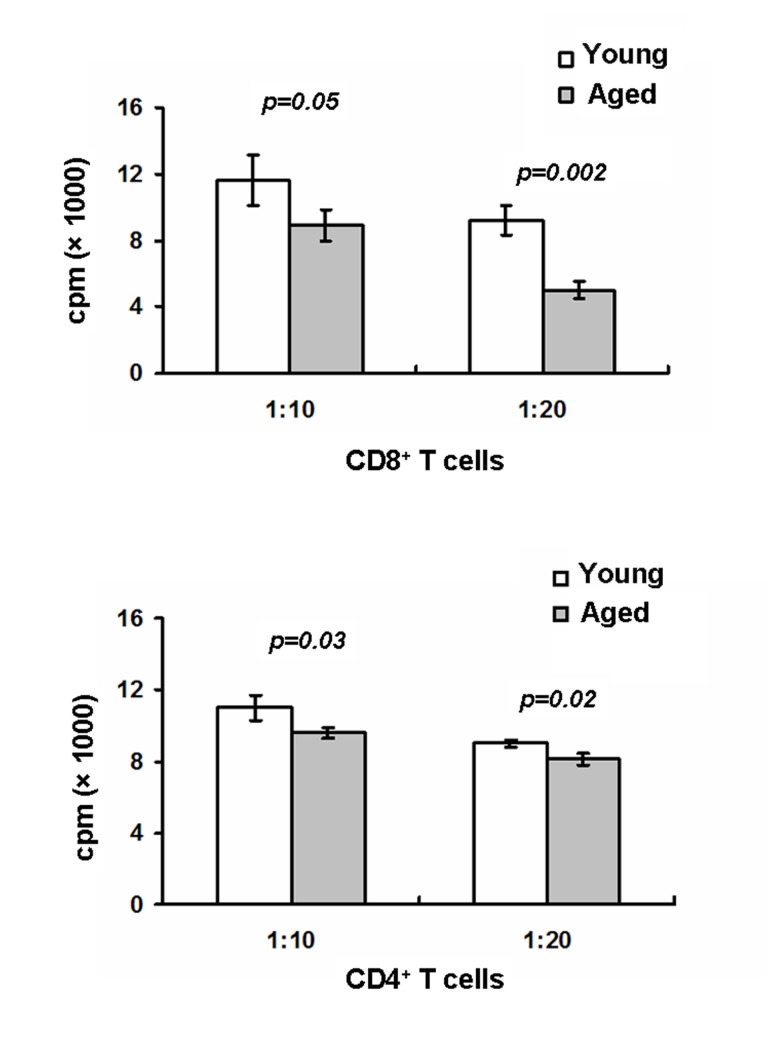
Aged mice exhibit defects in LC-mediated T cell proliferation LCs derived from aged and young mice were pulsed for 20 hr at 37°C with OVA protein (0.5mg/ml) then washed to remove excess OVA. LCs were first purified using AutoMACS with anti-MHCII-PE and anti-PE microbeads followed by a cell sorter. OT-I specific CD8 T cells purified from OT-I mice or OT-II specific CD4 T cells purified from OT-II mice were cultured with LCs for 72h. 1μCi of [^3^H] thymidine/well was added during the last16-18h. Cells were then harvested and radioactivity counted. Data are representative of two experiments.

LCs play a key role in mediating antigen-specfic T cell proliferation. Upregulation of MHCII and co-stimulatory molecules on LCs are required for T cell activation [30]. Given the in vitro downregulation of MHCII, CD80 and CD86 on aged LCs, we expected that aged LCs would have a reduced ability to active T cells. To test this, epidermal LCs were first pulsed with OVA protein and sorted LCs were then cultured with OVA-specific CD8^+^ OT-I or CD4^+^ OT-II T cells for 72h. As shown in Figure 4, aged LCs-mediated CD8^+^ OT-I cell and CD4^+^OT-II T cell proliferation were dramatically reduced compared to young LCs, Thus, our data further suggest the defective LC-mediated T cell activation in aged mice.

### Distinct miRNA expression profiles in aged epidermal LCs

**Figure 5:**
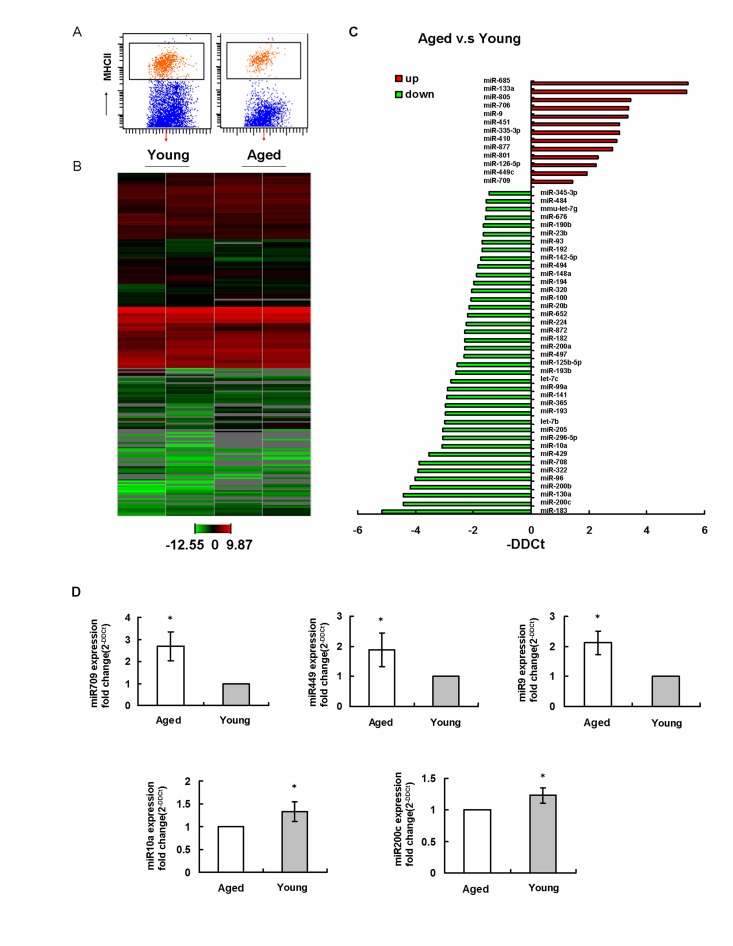
Aging regulates miRNA gene expression profiles in epidermal LCs (**A**) LCs were isolated using AutoMACS with anti-MHCII-PE and anti-PE microbeadsfollowed by a cell sorter. The purity was confirmed to be ~98%.(**B**) Expression profiles of miRNAs in young and aged LCs from two individual experiments. (**C**) miRNA expression in LCs was significantly up- and downregulated in aged mice compared to that in young mice. (**D**) Changed miRNAs in aging were confirmed by single TaqMan RT-PCR.

A recent study showed that deletion of miRNA precursor-processing enzyme Dicer in DC precursors interrupted the homeostasis and function of LCs. This suggested a key role of miRNAs in LC development and function [15]. Interestingly, miRNAs have also been shown to regulate the aging processes in different tissue and cells [31]. To test if the miRNAs are potentially involved in aged LCs development and function, the global miRNA expression profiles of sorted LCs from aged and young mice (Figure 5 A) were performed by TaqMan qPCR-based arrays (Figure 5 B). Using global normalization [32], we have identified 53 age-regulated miRNAs in LCs (fold change > 3), including 40 downregulated miRNAs and 13 upregulated miRNAs (Figure 5 C). Based on the miRNAs potentially linked to LCs development and function, we have further confirmed that miR-709, miR-449 and miR-9 were upregualated in aging, while miR-200c and miR-10a were downregulated in aging by using single TaqMan RT-PCR assays (Figure 5 D).

**Figure 6:**
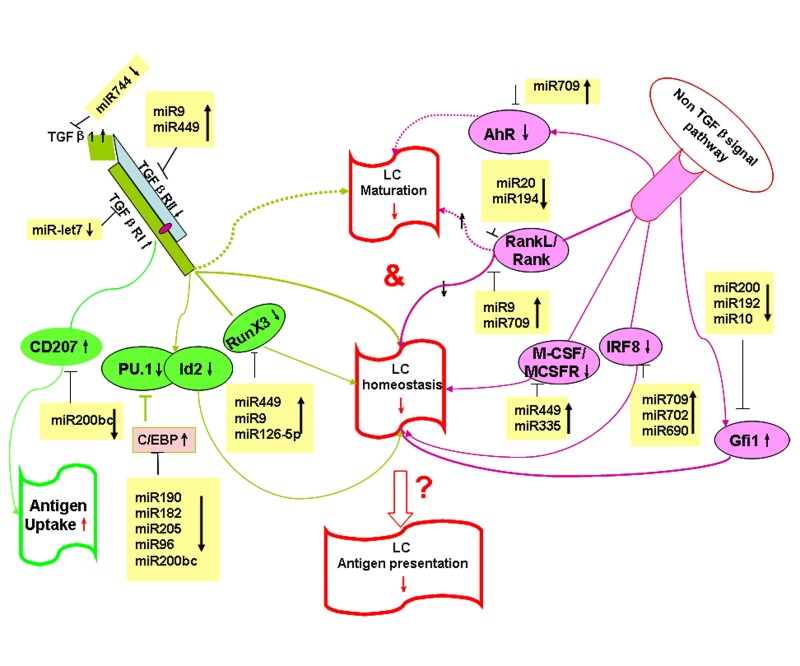
Hypothetical model: age-regulated miRNAs control LC development and function Aging regulated miRNAs potentially target TGF-β-dependent and non-TGF-β-dependent pathways that control LC development and function during aging.

To explore the potential targets and pathways that are regulated by aging-associated miRNAs in LCs, we used the Target Scan Mouse 6.2 database to predict miRNA targets related to LC homeostasis and function (Table 1). As shown in Table 1, Gfi1, IRF8, RunX3, C/EBP, TGFβRII, TGFβ1, AhR, RANKL, Langerin, and CSF1R are shown to regulate the homeostasis, development and function of epidermal LCs [27, 33-40], and are predictive targets by age-associated miRNAs. A hypothetical model of age-dependent miRNAs regulating LCs development and function is shown in Figure 6.

**Table 1:** Aging-regulated miRNAs potentially targets genes involved in LC development and function

miRNAs in aging	putative targets	function in LC	reference
miR709↑	RANK	LC development and homeostasis↓	49
	IRF8	LC development and homeostasis↓	29
	AhR	impair LC maturation	33
miR449↑	TGFβRII	LC development and homeostasis↓	32,46
	RunX3	LC development and homeostasis↓	30
	CSF1R	LC development and survival↓	35
miR9↑	TGFβRII	LC development and turnover↓	32,46
	RunX3	LC development and homeostasis↓	30
	RANK	LC development and homeostasis↓	49
miR10a↓	Gfi1	LC development and homeostasis↓	28
miR200c↓	C/EBP	LC differentiation↓	31
	Langerin	LC antigen uptake ↑	22, 23
	Gfi1	LC development and homeostasis↓	28
miR744↓	TGFβI	inhibit LC maturation	32,46
miR20b↓	RANKL	inhibit LC maturation	34
miR205↓	C/EBP	LC differentiation↓	31

## DISCUSSIONS

DCs are critical immune players involved in the induction of immunity and tolerance. Age-associated changes in DC development and function significantly compromise the immune system and directly impact both adaptive and innate immunity in the elderly [41]. LCs, part of the DC family, form a network within the epidermis where they play key roles in immunosurveillance for cutaneous malignancy, infection, allergy and autoimmune diseases [42]. We have demonstrated here that, in steady-state, the frequency of epidermal LCs is significantly reduced starting at 12 months old mice compared with that of young mice. This observation is in consistence with previous reports in mice [21]. Interestingly, LC survival and proliferation in the epidermis were not affected by aging. This finding suggests that the decreased LC density found in aged mice may result from a deficiency in bone marrow-derived LC progenitors, or that LC progenitors are less responsive to the chemokine and cytokine signals that are known to be required for the recruitment of LC progenitors into the skin [21, 43].

Effective migration of LCs into lymph nodes is necessary for LCs to induce T cell-mediated immune responses. Previous studies reported that migration of LCs in response to TNFa is significantly impaired in elderly subjects, but responses stimulated by similar exposure to IL-1β are unimpaired in the elderly [10, 44]. In contrast, Ogden et al. found that migration of LCs in response to a chemokine ligand, CCL19, was equivalent in both these groups [45]. Our data indicate that the migration of FITC-bearing LCs to skin draining LN was comparable between aged and young mice. However, the total LCs in draining LN was significantly decreased during aging (Figure 1 C), which could be related to reduced epidermal LCs. Interestingly, the ability of respiratory DCs to migrate to dLNs is compromised in aged mice with a decline in migration occurring as early as 6 months of age [9]. This suggests that different DC subsets may respond to aging differently.

The functional abilities of aged DCs in mice have primarily been generated using in vitro differentiated BMDCs. In some studies, BMDCs from aged mice had impaired maturation, antigen-presenting function, and reduced ability to stimulate T cell response [46, 47]. However, others have shown that the T cell priming ability of aged BMDCs was equivalent to their younger counterparts [6]. Whether the results generated from BMDCs can be extrapolated to LC functions in aged mice remains unclear. In our study, we found that LCs from aged mice appear to have a lower ability to up-regulate MHCII and co-stimulatory molecules including CD80 and CD86 compared to LCs from young mice after *in vitro* maturation. These molecules are known to play an important role in T cell priming. Thus, as expected, our data show that LCs from old mice are less effective in activating MHC class I-restricted CD8^+^T cells as well as MHC class II-restricted CD4^+^ T cells. This finding is consistent with previous findings in other aging DC subsets such as respiratory DCs and mucosal DCs [9, 48]. Reduced LC frequency in aged mice combined with their functionally defective of priming T cell immunity could contribute to age-related skin diseases.

Aging is a multifactorial process where deterioration of an organisms function is driven by distinct genetically encoded systems [49]. In recent years, more and more evidence is emerging that miRNAs are key players in the regulation of a wide range of biological functions in a variety of mammalian cell types, including immune cells [12, 50, 51], and that miRNAs have participated in the complex networks of cellular senescene and contribute to aging [31, 52-54]. The deletion of Dicer, an enzyme required for the processing of mature and functional miRNAs, provides a genetic test for the relevance of miRNAs in mammalian development. We (unpublished data) and others [15] recently found that Dicer deletion interrupted the homeostasis and function of LCs but not other DCs subsets, suggesting a specific role of miRNAs in LCs development and function. However, most individual miRNA effects on LCs development and function are unknown. We recently found miR-150 may regulate LC-mediated cross-presentation [55, 56]. Here, we identified specific age-related miRNA gene expression profiles in LCs, which could contribute to aged LCs homeostasis and function. TGF-β is well known for its essential role in LCs development and maturation [37, 57]. The importance of TGF-β signaling in LCs lineage decisions has been demonstrated in RunX3 knockout (KO) and Id2KO mice. Deletion of these two signaling molecules downstream of the TGFβR abolished LC development [35, 58]. Reciprocal roles for C/EBP and PU.1 in LC development have also been identified [36, 59]. miRNAs miR-449 and miR-9 potentially target TGFβ1, TGFβRI, TGFβRII, RunX3 and C/EBP, which are involved in TGF-β signaling (Figure 6). Thus, upregulated miR-449 and miR-9 in aged LCs could downregulate the TGF-β signaling pathway and block LC development. In addition, non-TGF-β signaling via CSF1/CSFR and IRF8 may also play an important role in LC homeostasis and development. IRFKO and CSFRKO mice both exhibit deficiencies in the frequency of LCs (Figure 6) [34, 40]. Interestingly, miR-709 and miR-449 also target IFR8 and CSF1R. Thus, upregualated miR-709 and miR-449 in aging LCs may downregulate the expression of IFR and CSFR, causing a deficiency in LCs development in aging mice. Recent reports indicated that Gfi1 plays a key role in LC homeostasis and Gfi1KO mice increased the frequency of LCs [33]. Gfi1 is a potential target of miR-200. Downregulated miR-200 in aged LCs may upregulate its target, Gfi1, which could then arrest LCs commitment. The RANKL/RANK system has been shown to play a key role in LCs homeostasis and maturation, and RANKL-deficient mice displayed a marked reduction of epidermal LCs [39, 60]. Interestingly, RANKL/RANK are putative targets of miR-9 or miR-20. Valladeau et al. previously reported that Langerin was the main molecular component of Birbeck granules, and that Langerin plays an important role in antigen uptake [27, 28]. Our miRNA array data showed that the expression level of miR-200 that potentially targets Langerin, was downregualted in aged LCs. This may account for the high expression of Langerin and the enhanced antigen-uptake ability in aging LCs. Overall, aging-regulated miRNAs could impair aged LCs development, maturation, and function by targeting different signaling pathways. To our knowledge, this is the first report to demonstrate that aging regulates miRNA gene expression in LCs.

In summary, our results demonstrate that the number and function of epidermal LCs are changed during aging development. Our comprehensive miRNA profile analyses on epidermal LCs uncover specific miRNA signatures in aged LCs, which could target LC-related signaling pathways and underlie impaired age-related LCs developmental and functional defects. Future work is needed to determine the role of specific age-associated miRNAs and their targets in LCs develop-ment and function, which will not only help for fully understanding aging LC biology, but also for LCs-related therapeutic strategies.

## METHODS

### Animals

Young (< 6 months) and aged (> 12 months) C57BL/6J mice, as well as OVA-specific MHCI-restricted and MHCII-restricted T cell receptor(TCR)-transgenic C57BL/6J mice (OT-I and OT-II) were purchased from Jackson Laboratory(Bar Harbor, ME), and were bred and housed in a specific pathogen-free barrier unit. Handing of mice and experimental procedures were in accordance with requirements of the Institutional Animal Care and Use Committee.

### Cell suspension preparations and Flow cytometry

Whole body skin from young (< 6 months) and aged (>12 months) mice was floated on dispase (2.5mg/ml) for 50 min in a 37°C incubator. The epidermis was separated and then digested with typsin (0.05%) and Dnase (0.1mg/ml) for 15 min in a shaking bath. Cells were filtered through a 70μm strainer to obtain single cell suspensions. Single-cell suspensions were incubated with anti-FcγRII/III (clone 2.4G2) and stained for surface and intracellular markers with the following antibodies: I-A/E(M5/114.15.2), CD45.2 (104), CD80 (16-10A1), CD86 (GL1), CD3e (145-2C11), CD4 (GK1.5), CD8 (53-6.7), EpCAM (G8.8), and Langerin (929F3.01) (purchased from eBioscience or Dendritics). Data were analyzed with a FACSAria™II (BD Bioscience) and Flowjo software. Apoptosis and proliferation assays were carried out by staining with Annexin V, 7-AAD (BD Bioscience) and Ki67 (SolA15) according to the manufacturer's instructions.

### Immunohistochemistry of epidermal sheets

Epidermis from ear skin was separated from the dermis according to previously published method [61], with minor modifications. Separated ear epidermis was blocked with staining buffer containing 1% FBS for 15 min and fixed with acetone at 4°C for 10min. They were then washed with PBS and stained overnight with CD3-FITC/IAb-PE in a 37°C incubator. Sheets were then placed on a slide and mounted under a coverslip with one drop of IMMU-MOUNT (Thermo scientific, Pittsburgh, PA). Specimens were viewed on a LEICA DMIRB microscope.

### Assay for phagocytosis and hapten-induced LC migration

Freshly separated epidermal cells were incubated with 0.25mg/ml FITC-Dextran for 45min at 4°C (control) or 37°C, and then stained with anti-MHCII and anti-CD45.2. The percentage of FITC^+^ cells in LCs was determined. For FITC-induced LC migration, mice were painted on the abdomen with 200μl 0.5%FITC in acetone/dibutylphthalate (1:1), and 24h later the inguinal LN were collected and then gently disrupted and digested with Dnase at 37°C in a shaking water bath for 20min. Single-cell suspensions were filtered through a 40μm strainer and were then stained with the following Abs to surface and intracellular markers: Langerin, CD8, EpCAM, and MHCII. Cells were analyzed by FACS and resulting data by Flowjo Software.

### In vitro OVA presentation

Epidermal cells suspension was prepared from ear and trunk skin. Cells were loaded with OVA protein (0.5mg/ml) for 20h in 37°C incubator. Cells were washed and cultured in with fresh medium without OVA for further culture overnight. Following culture the epidermal cells were first enriched by AutoMACS with anti-MHCII-PE and anti-PE microbeads (Miltenyi Biotec), followed by an additional sort through the AriaII cell sorter. Purity of the sorted cells was assessed by flow cytometry and confirmed to be ~98%. Spleen CD8^+^ T cells from OT-I mice and CD4^+^ T cells from OT-II mice were sorted and incubated in triplicate with epidermal LCs for 48h in round-bottom 96-well plates (ratio, 2.5×10^3^ LCs : 2.5×10^4^ T lymphocytes or 1.25×10^3^ LC : 2.5×10^4^ T lymphocytes) for 56h. 1μCi of [^3^H]thymidine was then added to each well and plates further incubated for 18h. Individual wells were then harvested and radioactivity counted.

### miRNA expression profiling and miRNA target analyses

Total RNA was collected in LCs from young (< 6 months-old) and aged (> 12 months old) mice by using the Qiagen miRNeasy Mini kit (Qiagen Inc, Germantown, MD) according to the manufacturer's procedure. miRNAs expression profiles were performed by TaqMan MicroRNA Assay rodent Set (Applied Biosystems, Foster, VA) on 7900HT Fast Real-Time PCR System (Applied BIosystem, Foster City, CA, USA), according to the manufacturer's recommended protocol. Raw cycle threshold (Ct) values were calculated using the SDS 2.3 and RQ manager 1.2 software (Applied Biosystems) applying automatic baseline and threshold settings. For global normalization, all the Ct values were imported into StatMiner® 4.2 (Integromics® Inc., Philadelphia, PA). The -ΔCt was calculated, and heatmap analysis was performed with hierarchical clustering. Target prediction was performed using the open source program targetscan (www.targetscan.org).

### Real-time RT-PCR for miRNAs expression

The expression levels of miRNAs were examined using the Applied Biosystems TaqMan MicroRNA Assay kit (Applied Biosystems, Foster, CA) according to the manufacturer's instructions. The kit uses gene-specific stem-loop reverse transcription primers and TaqMan probes to detect mature miRNA transcripts. PCR amplification was carried out on the Applied Biosystems 7900 Real-time PCR system. The assay was run in triplicate for each case to allow for assessment of technical variability. SnoRNA135 was used as endogenous control. Relative quantitations using the ∆∆Ct method in aged versus young mice were carried out and fold changes were calculated by 2^−∆∆Ct^ for each miRNA gene.

### Statistical analysis

Statistical analysis was performed with Prism5.0 (Graphpad Software). The two-tailed Student t test was used. Differences were considered statistically significant when values of p < 0.05 were obtained.
